# Supplementary material for: In mitosis integrins reduce adhesion to extracellular matrix and strengthen adhesion to adjacent cells
Source: Nat Commun. 2023 Apr 14;14:2143. doi: 10.1038/s41467-023-37760-x (PMC10104879; doi:10.1038/s41467-023-37760-x)
Supplement: Supplementary file 1 — Supplementary Information [file 41467_2023_37760_MOESM1_ESM.pdf]

Supplementary Information

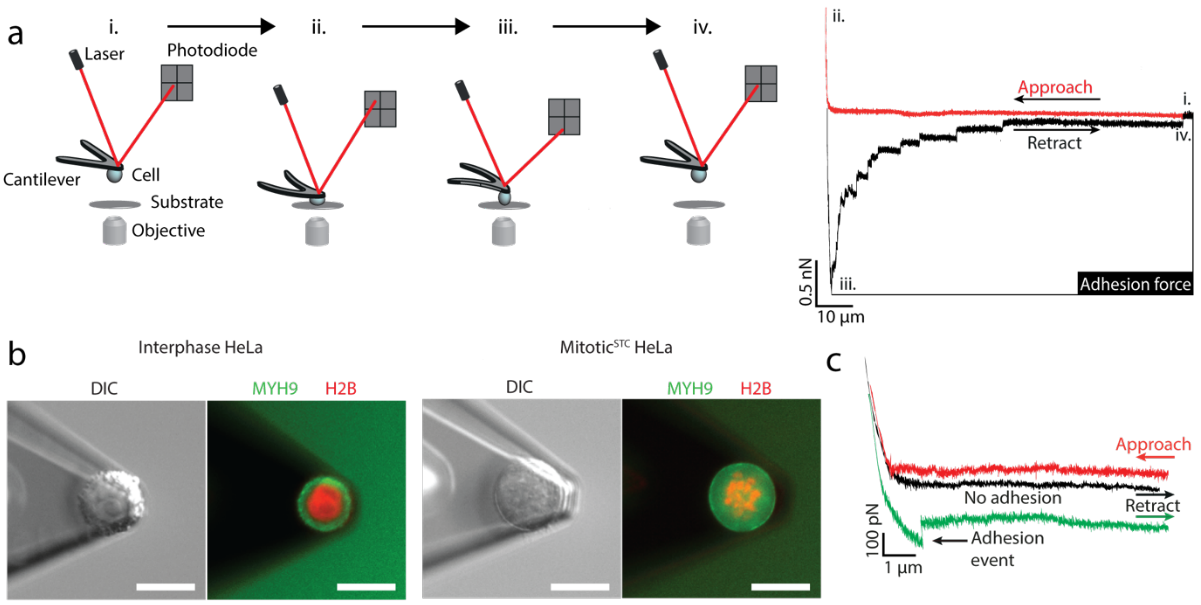

Supplementary Fig. 1 | AFM-based single-cell force spectroscopy (SCFS) to study adhesion initiation and strengthening of cells in interphase or mitosis. a, AFM-based SCFS. **i**, A single cell is attached to a tipless concanavalin A (ConA)-coated cantilever and incubated on the cantilever for 10 min to ensure firm binding. **ii**, The cantilever-bound cell is brought into contact with the substrate. After a given contact time, the cantilever-bound cell is retracted (**iii** and **iv**) from the substrate until the cell is fully detached from the substrate to measure the adhesion force between the cell and the substrate. The substrate is either a Matrigel- or purified ECM protein-coated support, or cells spread on Matrigel-coated supports. During the adhesion experiments, the cantilever deflection is recorded and displayed in a force-distance curve. The retraction force-distance curve (black) records the adhesion force of the probed cell as the maximum downward deflection, which represents the highest force the cell can bear before being detached from the substrate. In total, in this work, 7’570 force-distance curves were recorded using single cells as probes and to detect cell adhesion forces. **b**, Differential interference contrast (DIC) and wide field fluorescence microscopy images to monitor the cell morphology of interphase (left) and mitotic^{STC} (right) HeLa cells expressing MYH9-GFP and H2B-mCherry, adhering to a concanavalin A-coated cantilever ($n = 3$ independent experiments). Scale bars, 20 µm. **c**, Exemplary force-distance curve detecting the (un-)binding of single integrins or cadherins. To record single integrin/cadherin (un-)binding events with SCFS, cells are approached (red curve) to the substrate of interest until a contact force of ~150 pN was reached and immediately retracted, resulting in a contact time of ~120 ms between cell and substrate or ~150 ms between two cells. Representative force-distance curves showing either no adhesion event (black) or a single unbinding event (green curve). Binding probabilities are calculated as the ratio of force-distance curves showing an unbinding event and the total number of force-distance curves acquired for each cantilever-bound cell. A total of 39’245 force-distance curves were recorded for all cantilever-bound cells to detect the (un-)binding of single integrins and cadherins.

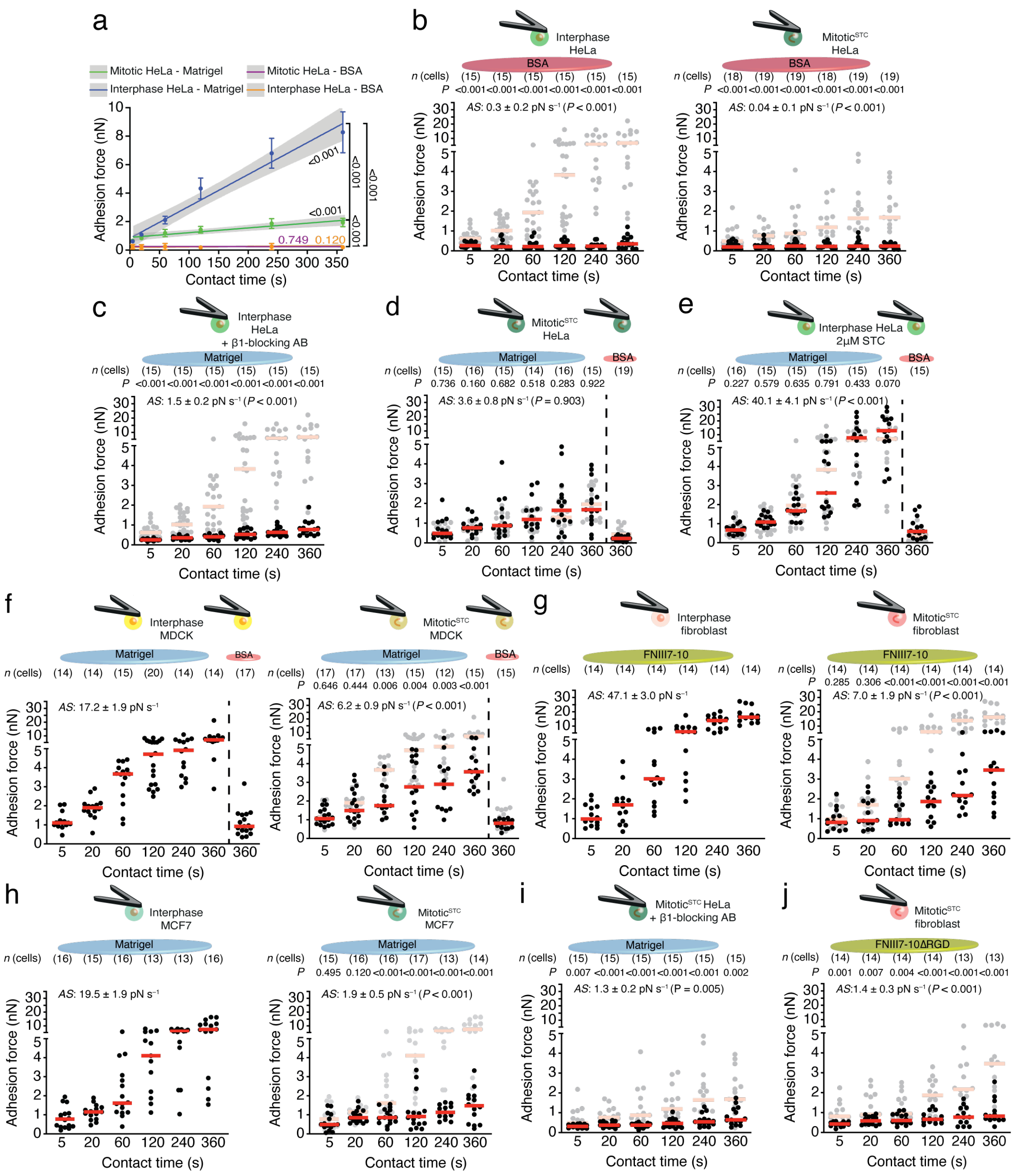

Supplementary Fig. 2 | Interphase and mitotic cells initiate and strengthen integrin-mediated adhesion to ECM proteins. a, Linear fits (lines) with 95% confidence intervals (grey) of adhesion forces to determine the adhesion strengthening rate (AS) of interphase or mitotic^{STC} HeLa cells to Matrigel or BSA (**Fig. 1a** and **Supplementary Fig. 2b;** $n \geq 15$ cells per condition and contact time). Dots represent mean adhesion force (±SEM). *P*-values on fits test slope deviations from 0 and on bars compare whether slopes are different (two-tailed extra sum of squares F-test). **b**, Adhesion forces of interphase (left) or mitotic^{STC} (right) to BSA and to Matrigel in grey as reference (**Fig. 1a**). **c**, Adhesion forces of interphase HeLa cells incubated with β1 integrin blocking-antibodies (clone AIIB2) to Matrigel and of unperturbed HeLa cells (**Fig. 1a**) in grey as reference. **d**, Adhesion forces of mitotic^{STC} HeLa cells to Matrigel or BSA and of untreated mitotic HeLa cells to Matrigel in grey as reference (**Fig. 1a**). **e**, Adhesion forces of interphase HeLa cells in the presence of 2 µM STC to Matrigel or BSA and of untreated interphase HeLa cells (**Fig. 1a**) in grey as reference. **f-h**, Adhesion forces of interphase (left)

or mitotic^{STC} (right) MDCK cells (**f**), fibroblasts (**g**) or MCF7 cells (**h**) to given substrates and of respective interphase cells as reference in grey. **i**, Adhesion forces of HeLa cells in the presence of AIIB2 to Matrigel and untreated mitotic^{STC} HeLa cells (**Fig. 1a**) in grey as reference. **j**, Adhesion forces of mitotic^{STC} fibroblast cells to fibronectin fragment FNIII7-10ΔRGD and to FNIII7-10 as reference in grey. **b-j,** Dots represent adhesion forces of single cells, red bars medians and (*n*) the number of tested cells. AS were determined like in a with the *P*-value comparing the *AS*-value to the reference data set. 'Mitotic^{STC}' indicates that mitotic cells were enriched by STC (Methods). Given *P*-values calculated using a two-tailed Mann-Whitney test compare displayed adhesion forces with reference data and comparing *AS*-values to given reference data were calculated by two-tailed extra sum of squares F-tests.

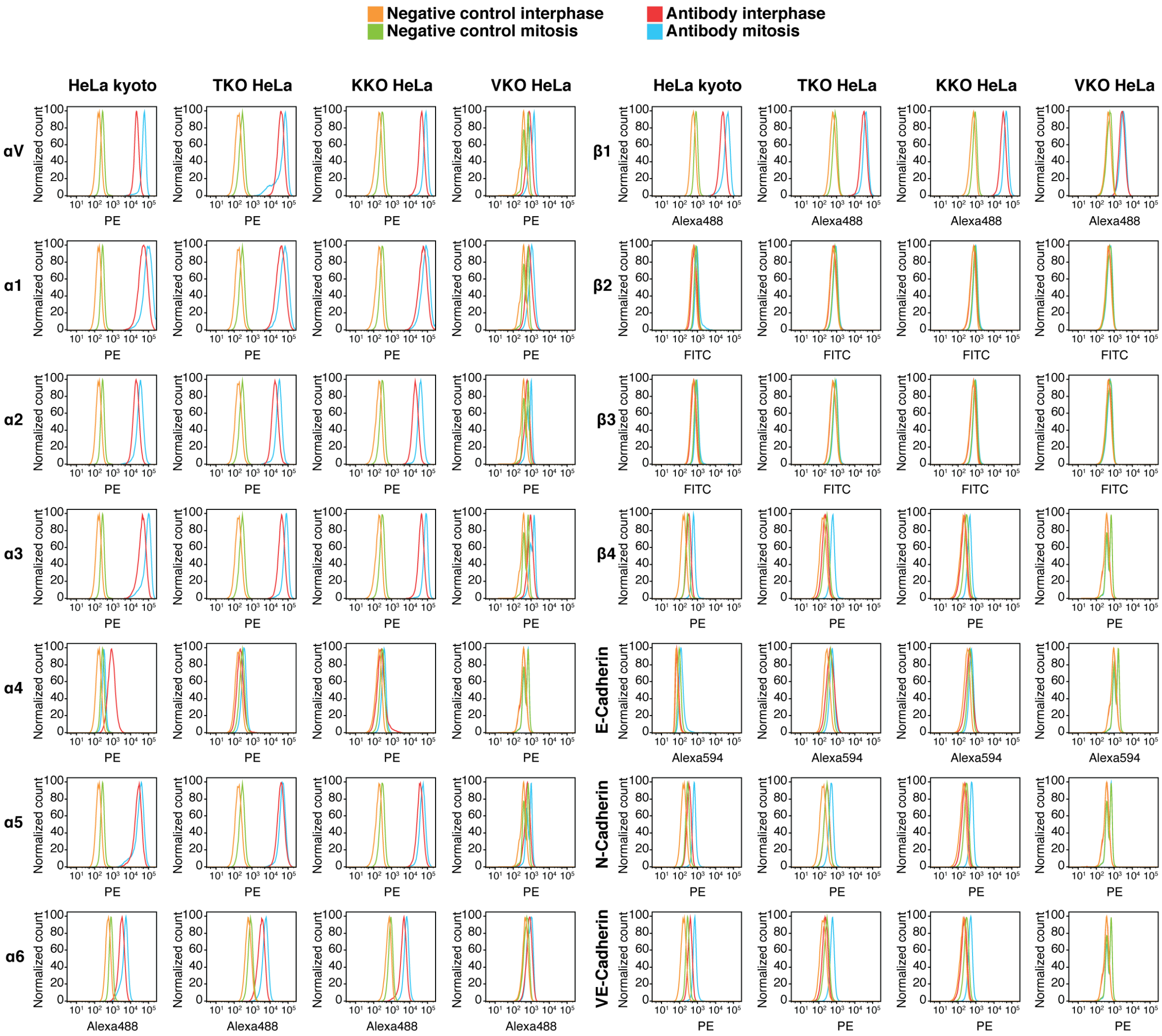

Supplementary Fig. 3 | Verification of adhesome depletion and characterization of integrin and cadherin expression of engineered HeLa cell lines. Flow cytometry analysis of HeLa (Kyoto), talin1/2-depleted (TKO), kindlin1/2-depleted (KKO HeLa) HeLa (Kyoto) and vinculin-depleted (VKO) HeLa MYH9-GFP H2B-mCherry cells for expression levels of given integrin subunits as well as E-, N- and VE-cadherin. Normalized histograms of fluorescence intensities for interphase or mitotic^{STC} HeLa cells stained with antibodies against the given integrin subunit or cadherin are shown. Negative controls were unstained cells. 50’000 cells were analyzed for each condition.

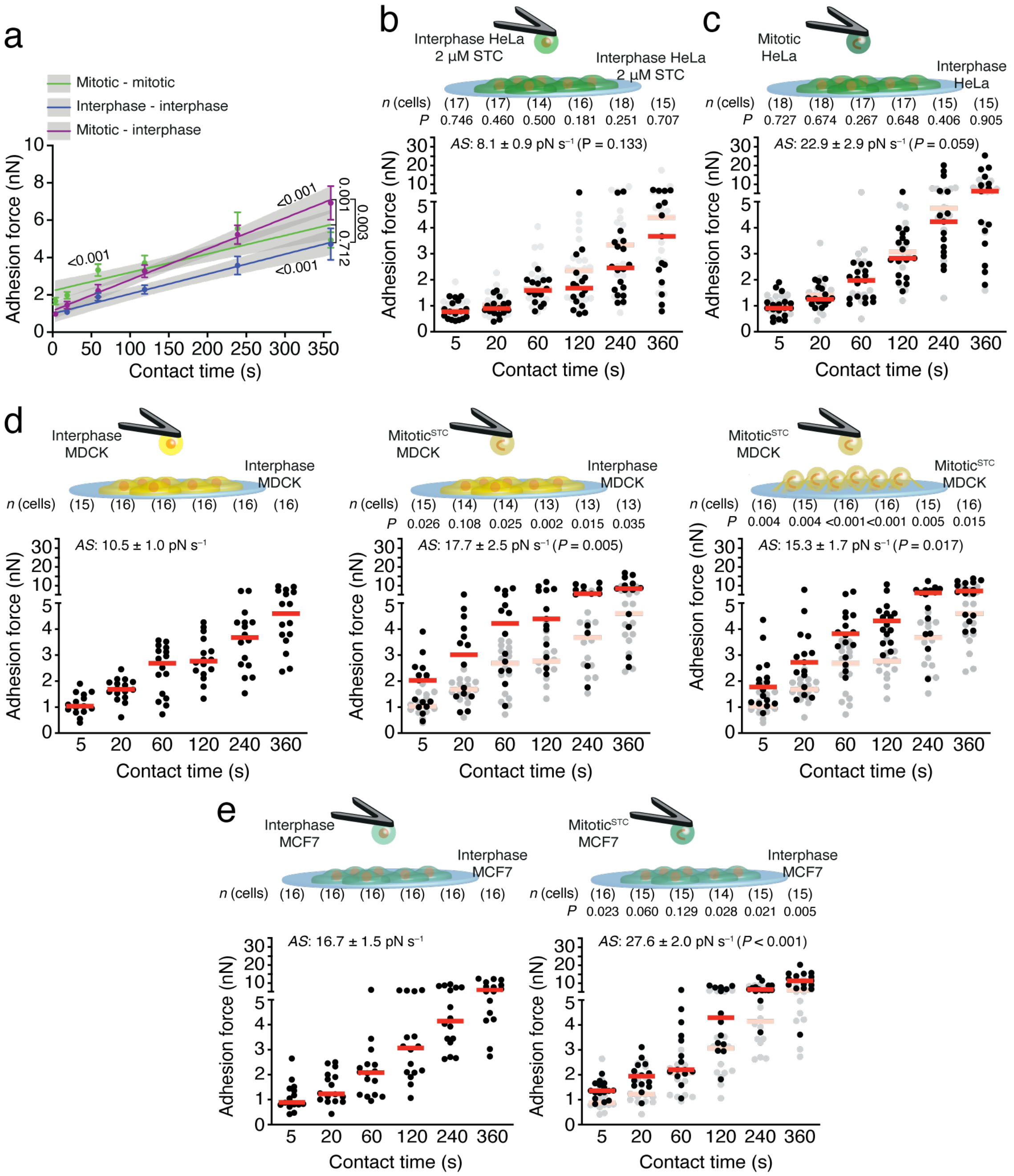

Supplementary Fig. 4 | Increased cell-cell adhesion of mitotic cells is maintained across cell lines. a, Adhesion strengthening over time is determined as a linear regression of adhesion forces for all contact times for given conditions (**Fig. 1g;** $n \geq 12$ cells per condition and contact time). Dots represent means (±SEM) of adhesion forces. Lines depict fit and grey areas their 95% confidence intervals. *P*-values on fits test slope deviations from 0 and on bars compare whether slopes are different (two-tailed extra sum of squares F-test). **b**, Cell-cell adhesion forces between two interphase HeLa cells in the presence of 2 µM STC (**Fig. 1g**). Dots represent adhesion forces of single cells, red bars median values and (*n*) the number of tested cells per condition. As reference adhesion forces between untreated interphase HeLa cells are given (**Fig. 1g**). *AS*-values give the adhesion strengthening rate as the slope (±SE) of a linear fit through adhesion forces for all contact times with the *P*-value comparing the *AS*-value to that of the reference data set. **c**, Adhesion forces between an untreated mitotic and an interphase HeLa cell. Data representation as in **b**. As reference, cell-cell adhesion forces between an interphase and mitotic^{STC} HeLa cells are given in grey (**Fig. 1g**). **d**, Cell-cell adhesion forces between two interphase (**left**), an interphase and a mitotic^{STC} (**middle**) and two mitotic^{STC} (**right**) MDCK cells. Data representation as in **b**. Cell-cell adhesion forces between two interphase MDCK cells are given

as reference in grey for comparison in the middle and right panel. **e**, Adhesion forces between two interphase (**left**) or an interphase and a mitotic^{STC} (**right**) MCF7 cell. Data representation as in **b**. Adhesion forces between two interphase MCF7 cells are given as reference in grey. **b-e,** Mitotic^{STC} cells were enriched by 2 μM STC for 12 h prior to and incubated with STC throughout the experiments. *P*-values comparing adhesion forces were calculated using two-tailed Mann-Whitney tests and compare displayed adhesion forces with given reference condition. *P*-values comparing *AS*-values were calculated by a two-tailed extra sum of squares F-test and compare *AS*-values of given and reference data.

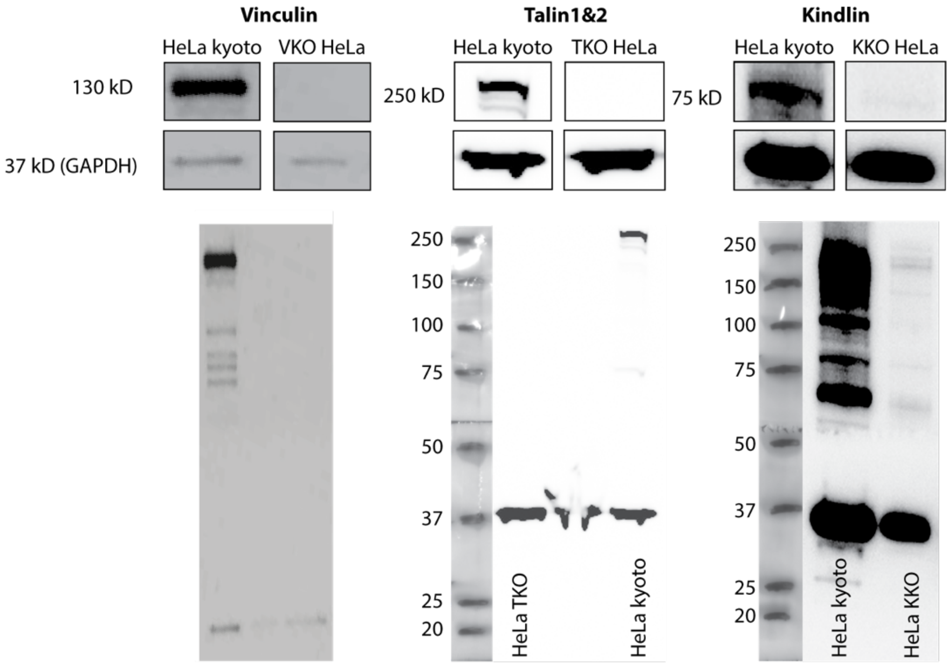

Supplementary Fig. 5 | Verification of adhesome protein depletion in HeLa cell lines. Cell lysates of control HeLa cells and HeLa cells depleted from vinculin (VKO), talin1/2 (TKO) or kindlin1/2 (KKO) were immunoblotted (*n* = 1). Wildtype HeLa cells were used as positive controls, GAPDH was used as loading control.

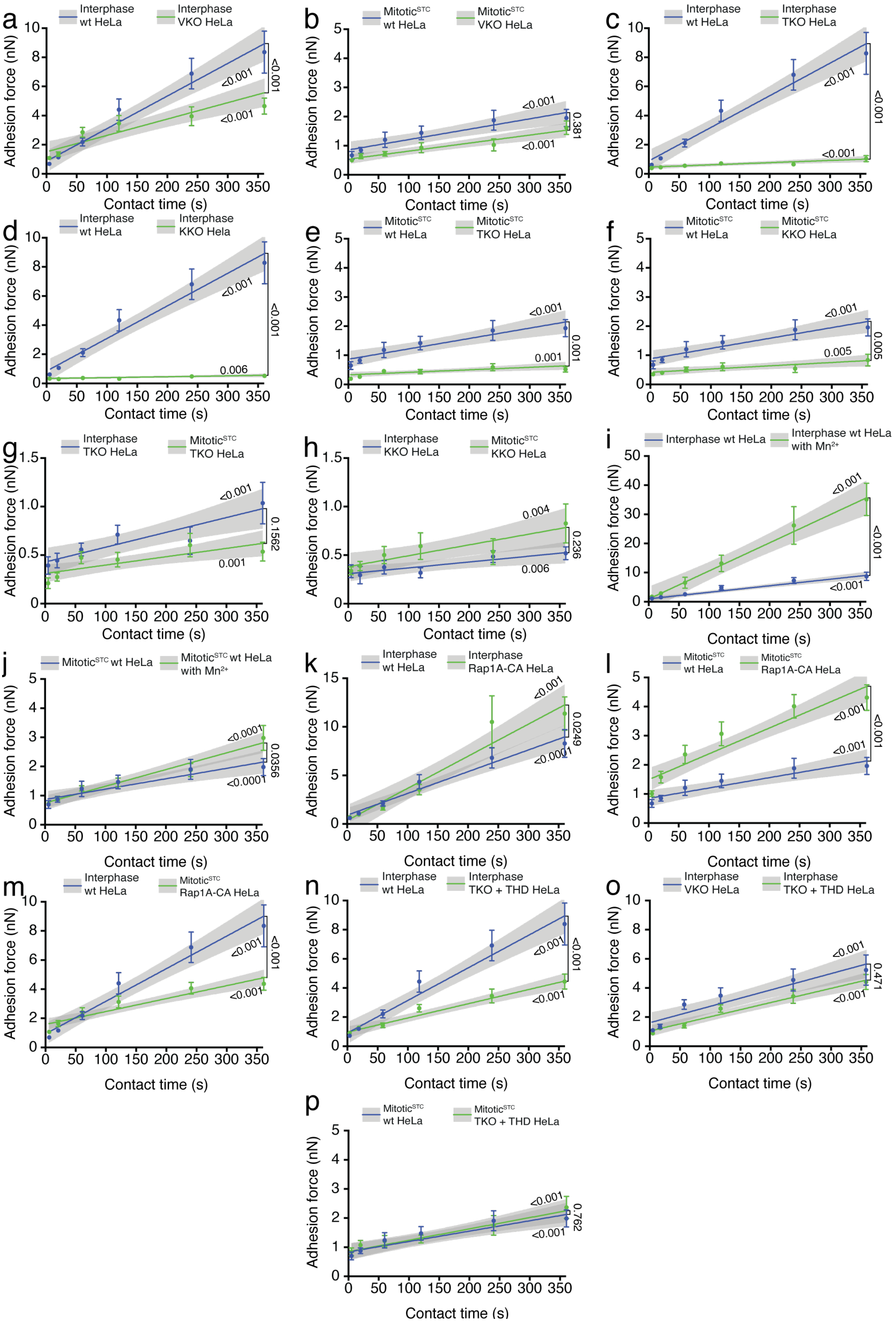
a
Interphase wt HeLa
Interphase VKO HeLa
<0.001
<0.001
<0.001
b
MitoticSTC wt HeLa
MitoticSTC VKO HeLa
<0.001
<0.001
0.381
c
Interphase wt HeLa
Interphase TKO HeLa
<0.001
<0.001
<0.001
d
Interphase wt HeLa
Interphase KKO Hela
<0.001
0.006
<0.001
e
MitoticSTC wt HeLa
MitoticSTC TKO HeLa
<0.001
0.001
0.001
f
MitoticSTC wt HeLa
MitoticSTC KKO HeLa
<0.001
0.005
0.005
g
Interphase TKO HeLa
MitoticSTC TKO HeLa
<0.001
0.001
0.1562
h
Interphase KKO HeLa
MitoticSTC KKO HeLa
0.004
0.006
0.236
i
Interphase wt HeLa
Interphase wt HeLa with Mn2+
<0.001
<0.001
<0.001
j
MitoticSTC wt HeLa
MitoticSTC wt HeLa with Mn2+
<0.0001
<0.0001
0.0356
k
Interphase wt HeLa
Interphase Rap1A-CA HeLa
<0.001
<0.001
0.0249
l
MitoticSTC wt HeLa
MitoticSTC Rap1A-CA HeLa
<0.001
<0.001
<0.001
m
Interphase wt HeLa
MitoticSTC Rap1A-CA HeLa
<0.001
<0.001
<0.001
n
Interphase wt HeLa
Interphase TKO + THD HeLa
<0.001
<0.001
<0.001
o
Interphase VKO HeLa
Interphase TKO + THD HeLa
<0.001
<0.001
0.471
p
MitoticSTC wt HeLa
MitoticSTC TKO + THD HeLa
<0.001
<0.001
0.762
Adhesion force (nN)
Contact time (s)

Supplementary Fig. 6 | Intracellular adaptor proteins affects adhesion initiation and strengthening of HeLa cells to Matrigel. a-p, Comparison of cell-ECM adhesion strengthening of given cell lines in the given cell cycle state to Matrigel (data taken from **Fig. 1a, Fig. 3, Fig. 5** and **Fig. 6;** $n \geq 12$ cells per condition and contact time). For each condition a linear regression of adhesion forces for all contact times determined the adhesion strengthening over time. Dots represent means (±SEM) of adhesion forces at given contact time. Lines depict the fit and the grey area the 95% confidence interval of the fit. *P*-values on fits test whether slopes deviate from 0. *P*-values on bars compare whether slopes of two fits are significantly different (two-tailed extra sum of squares F-test).

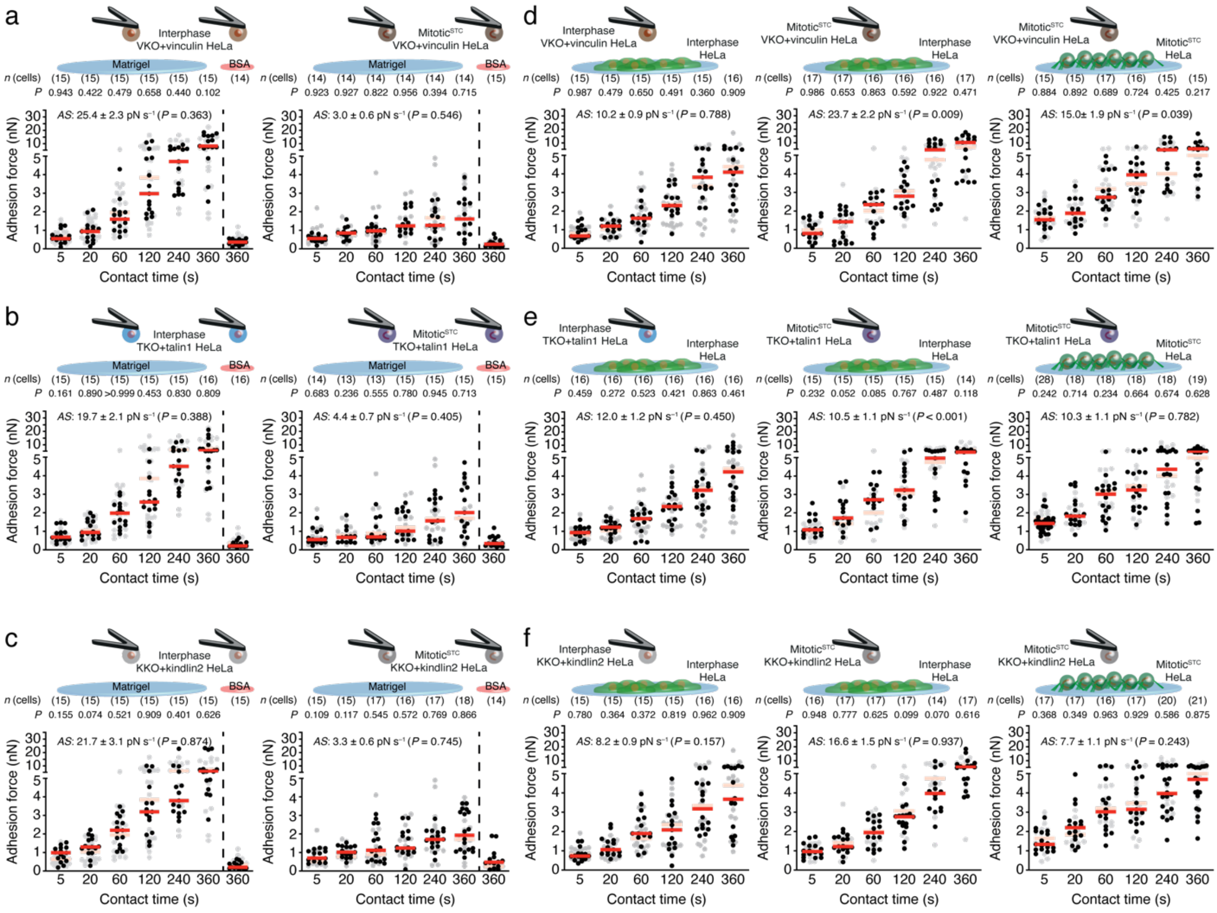

Supplementary Fig. 7 | Re-expression of specific adaptor proteins in KO HeLa cell lines recovers adhesion defects. a-c, Cell-ECM adhesion forces to Matrigel or BSA of (left) interphase and (right) mitotic^{STC} knock-out HeLa cells re-expressing the depleted protein adhesome vinculin (VKO+vinculin) (**a**), talin1 (TKO+talin1) (**b**) or kindlin2 (KKO+kindlin2) (**c**) after given contact times. Dots represent adhesion forces of single cells, red bars median values and (*n*) the number of tested cells per condition. As reference, cell-ECM adhesion forces of control HeLa cells are given in grey (**Fig. 1a**). *AS*-values give the adhesion strengthening rate as the slope (±SE) of a linear fit through adhesion forces for all contact times with the *P*-value comparing the *AS*-value to that of the reference data set. *P*-values compare the displayed data with the reference data given. **d-f**, Cell-cell adhesion forces between VKO+vinculin (**d**), TKO+talin1 (**e**) or KKO+kindlin2 (**f**) HeLa cells and control HeLa cells spread on a Matrigel coated substrate after given contact times. Panels show adhesion forces of respective interphase re-expressing and interphase control (**left**), mitotic^{STC} re-expressing and interphase control (**middle**) or mitotic^{STC} re-expressing and mitotic^{STC} control (**right**) HeLa cells. As reference cell-cell adhesion forces established between two control HeLa cells for the respective condition is given in grey (**Fig. 1g**). Data representation as in **a**. *P*-values compare displayed data with given reference data. Mitotic cells were enriched by 2 µM STC for 12 h prior to and incubated with STC throughout the experiments. *P*-values comparing adhesion forces with given reference condition were calculated using two-tailed Mann-Whitney tests and comparing *AS*-values to given reference data were calculated by two-tailed extra sum of squares F-tests.

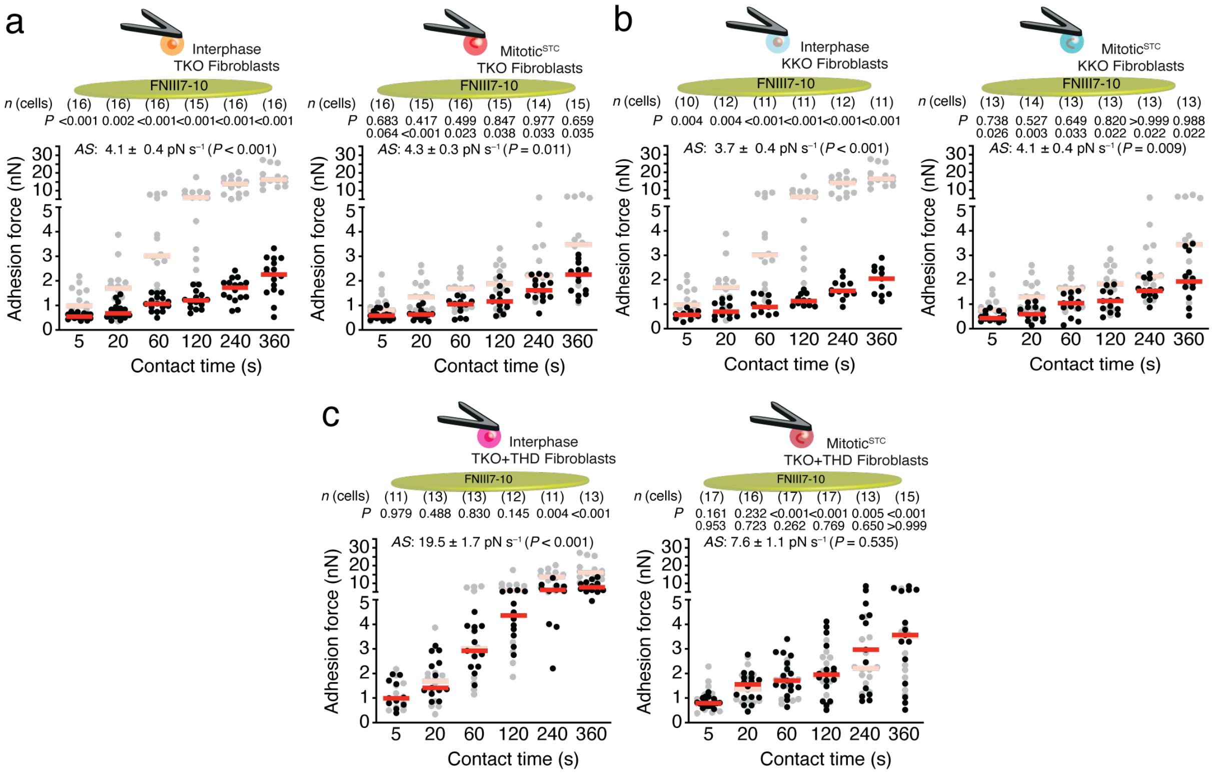

Supplementary Fig. 8 | Kindlin and talin are essential for mitotic cell-ECM adhesion in fibroblasts. Cell-ECM adhesion forces of interphase (**left**) or mitotic^{STC} (**right**) TKO (**a**), KKO (**b**) or TKO+THD (**c**) fibroblasts to FNIII7-10 at given contact times. Adhesion forces of wild type interphase or mitotic^{STC} fibroblasts to FNIII7-10 are given as reference in grey (**Supplementary Fig. 2g**). Dots represent adhesion forces of single cells, red bars median values and *n* (cells) number of tested cells per condition. *AS*-values give the adhesion strengthening rate as the slope (±SE) of a linear fit through adhesion forces for all contact times with the *P*-value comparing the *AS*-value to that of the reference data set. If only one row P-values are given they compare given and reference data. Otherwise, top row *P*-values compare mitotic^{STC} adhesion forces with respective interphase adhesion forces and bottom row *P*-values compare given and reference adhesion forces. 'Mitotic^{STC}' indicates that mitotic cells were enriched by 2 µM STC for 12 h prior to and incubated with STC throughout the experiments. *P*-values were calculated using two-tailed Mann-Whitney tests and *P*-values comparing *AS*-values were calculated by a two-tailed extra sum of squares F-test.

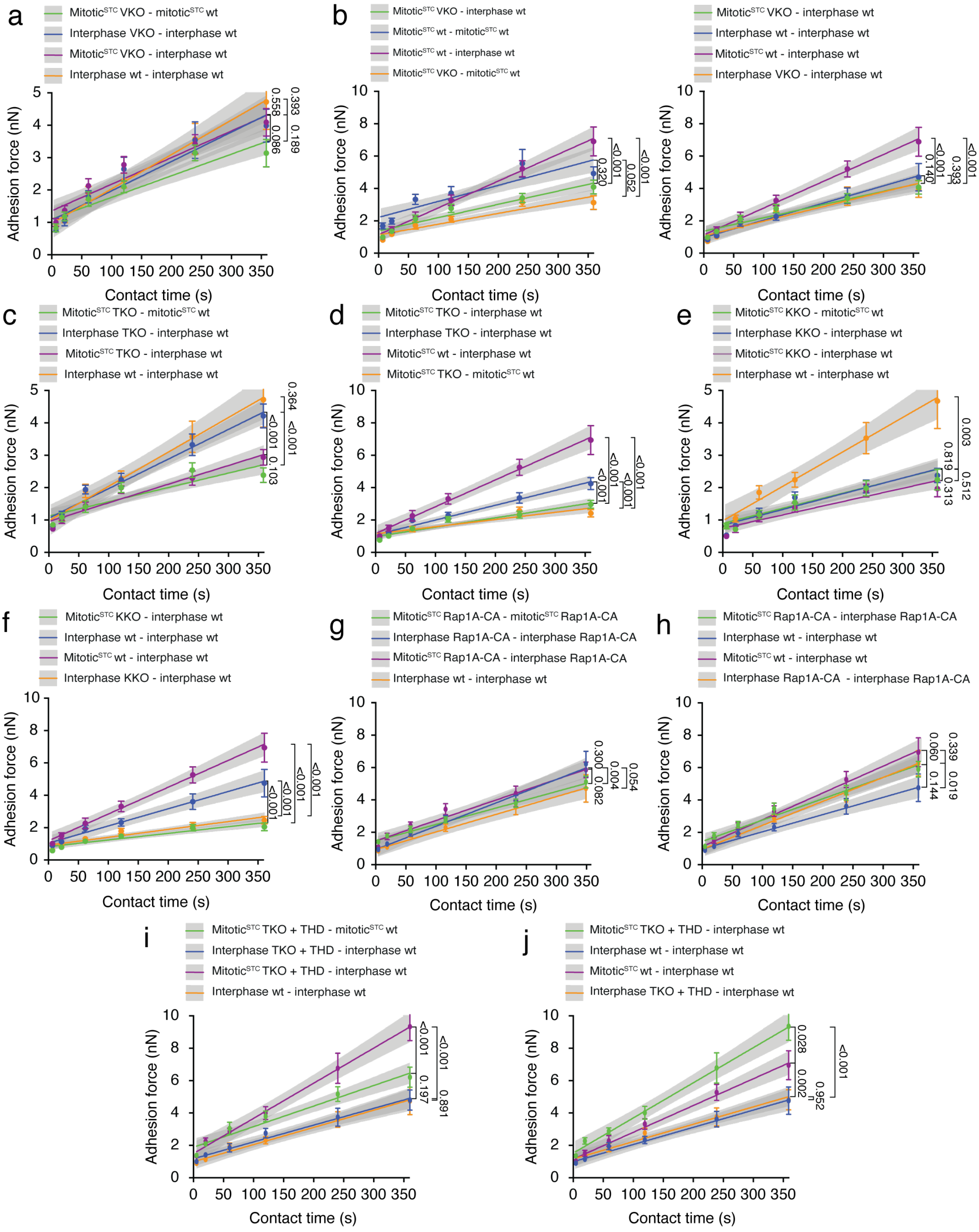

Supplementary Fig. 9 | Mitotic cell-cell adhesion strengthening depends on distinct adhesome proteins. a-j, Comparison of cell-cell adhesion strengthening of given cell lines in the given cell cycle state (data taken from **Fig. 1g**, **Fig. 4** and **Fig. 7**; $n \geq 12$ cells per condition and contact time). For each condition a linear regression of adhesion forces for all contact times determined the adhesion strengthening over time. Dots represent means (±SEM) of adhesion forces at given contact time. Lines depict the fit and the grey area the 95% confidence interval of the fit. *P*-values on bars compare whether slopes of two fits are significantly different (two-tailed extra sum of squares F-test).

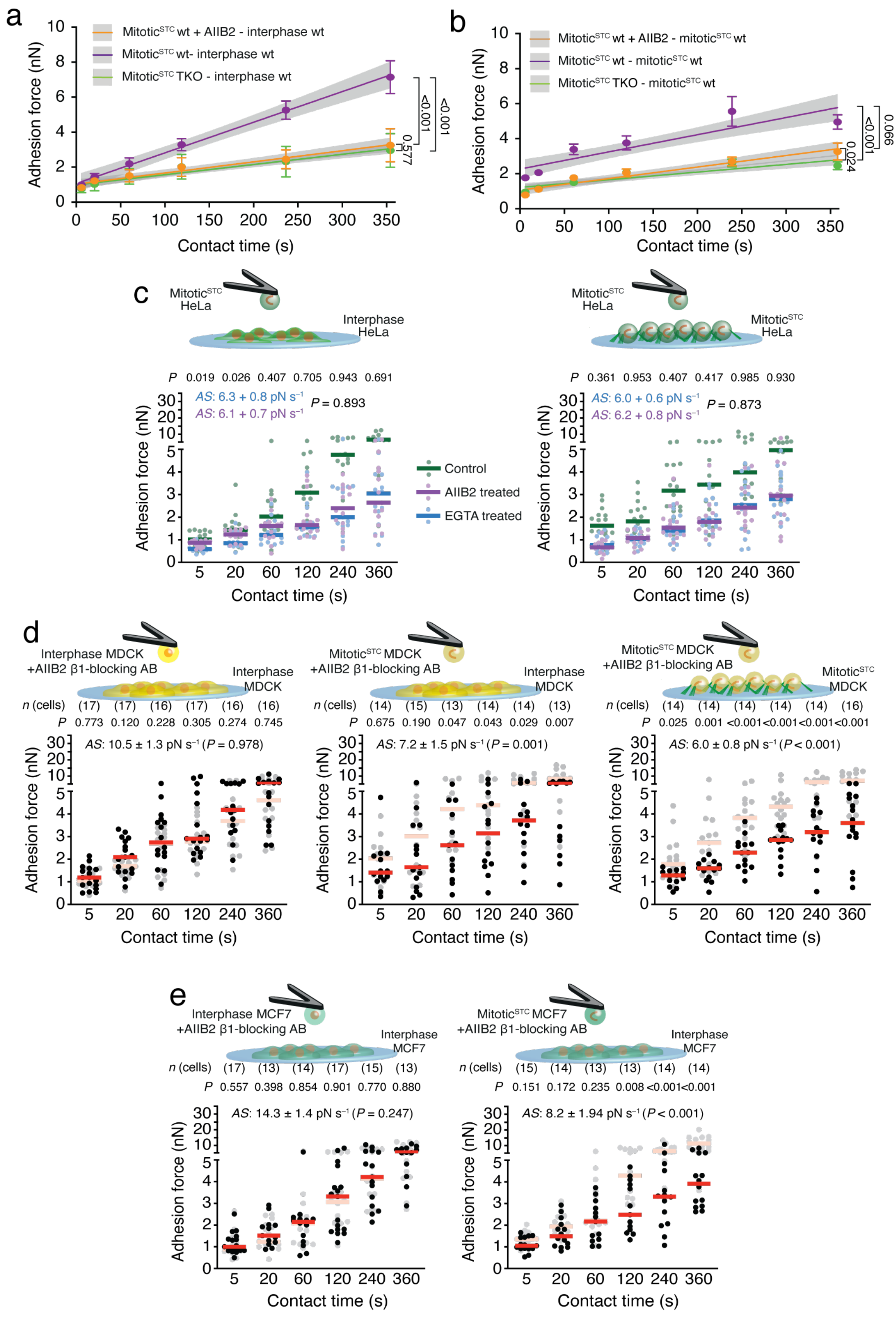

Supplementary Fig. 10 | Treatment with β1 integrin blocking antibody AIIB2 impairs mitotic cell-cell adhesion. a-b, Linear fits (lines) with 95% confidence intervals (grey) of cell-cell adhesion forces to determine the adhesion strengthening rate (AS) of given cell lines in the given cell cycle state (data taken from **Fig. 1g** and **Fig. 8b;** *n* ≥ 15 cells per condition and contact time). Dots represent means

(±SEM) of adhesion forces at given contact time. *P*-values on bars compare slopes of two fits (two-tailed extra sum of squares F-test). **c**, Adhesion forces of mitotic^{STC} HeLa cells, incubated with integrin subunit β1 blocking-antibody clone AIIB2 (purple) or EGTA (blue) to interphase (left) or mitotic^{STC} (right) HeLa cells after given contact times. Dots represent adhesion forces between single cantilever-bound and substrate-spread cells and bars their median. **d**, Cell-cell adhesion forces between two interphase (**left**), an interphase and a mitotic^{STC} (**middle**) or two mitotic^{STC} (**right**) MDCK cells, incubated with integrin subunit β1 blocking-antibody clone AIIB2 after given contact times. Dots represent adhesion forces between single cantilever-bound and substrate-spread cells, red bars median values and *n*(cells) the number of cells tested. As reference cell-cell adhesion forces established between two untreated MDCK cells in the respective condition are given in grey (**Supplementary Fig. 4d**). *P*-values compare adhesion forces of given and the reference data. *AS*-values give the adhesion strengthening rate as the slope (±SE) of a linear fit through adhesion forces for all contact times with the *P*-value comparing the *AS*-value to that of the reference data set. **e**, Cell-cell adhesion forces between two interphase MCF7 cells (**left**) or an interphase and a mitotis^{STC} MCF7 cell (**right**), incubated with integrin subunit β1 blocking-antibody clone AIIB2 after given contact times. Adhesion forces between untreated MCF7 cells in the respective condition is given in grey as reference. Data representation as in **d**. Mitotic cells were enriched by 2 µM STC 12 h prior to and throughout the experiments. *P*-values comparing adhesion forces were calculated using two-tailed Mann-Whitney tests and comparing *AS*-values to given reference data were calculated by two-tailed extra sum of squares F-tests.

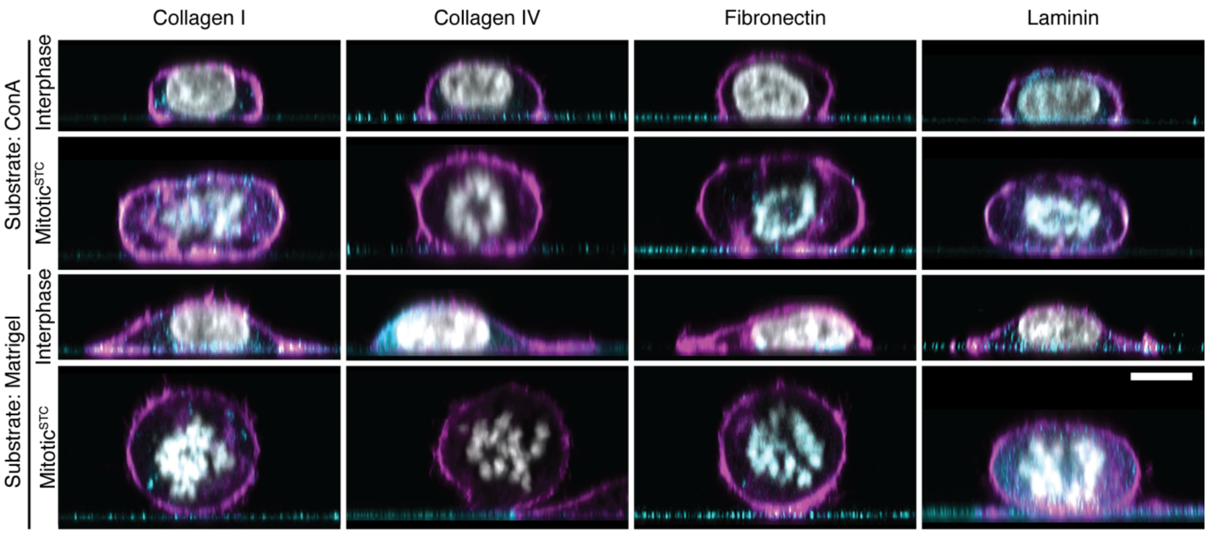

Supplementary Fig. 11 | Interphase or mitotic HeLa cells seeded on ConA or Matrigel show no collagen I, collagen IV, fibronectin and laminin on their surface. Representative orthogonal views (n = 5 cells) of interphase (top row) or mitotic^{STC} (second row) HeLa cells seeded on ConA for 30 min, interphase HeLa cells seeded on Matrigel for 2 h (third row), or HeLa cells seeded on Matrigel and arrested in mitosis by STC for 12 h (bottom row). Cells are labeled for collagen I (far left column), collagen IV (second column), fibronectin (third column) or laminin (right column) in cyan, actin in magenta and for DNA in white (Methods). Scale bar, 10 µm.

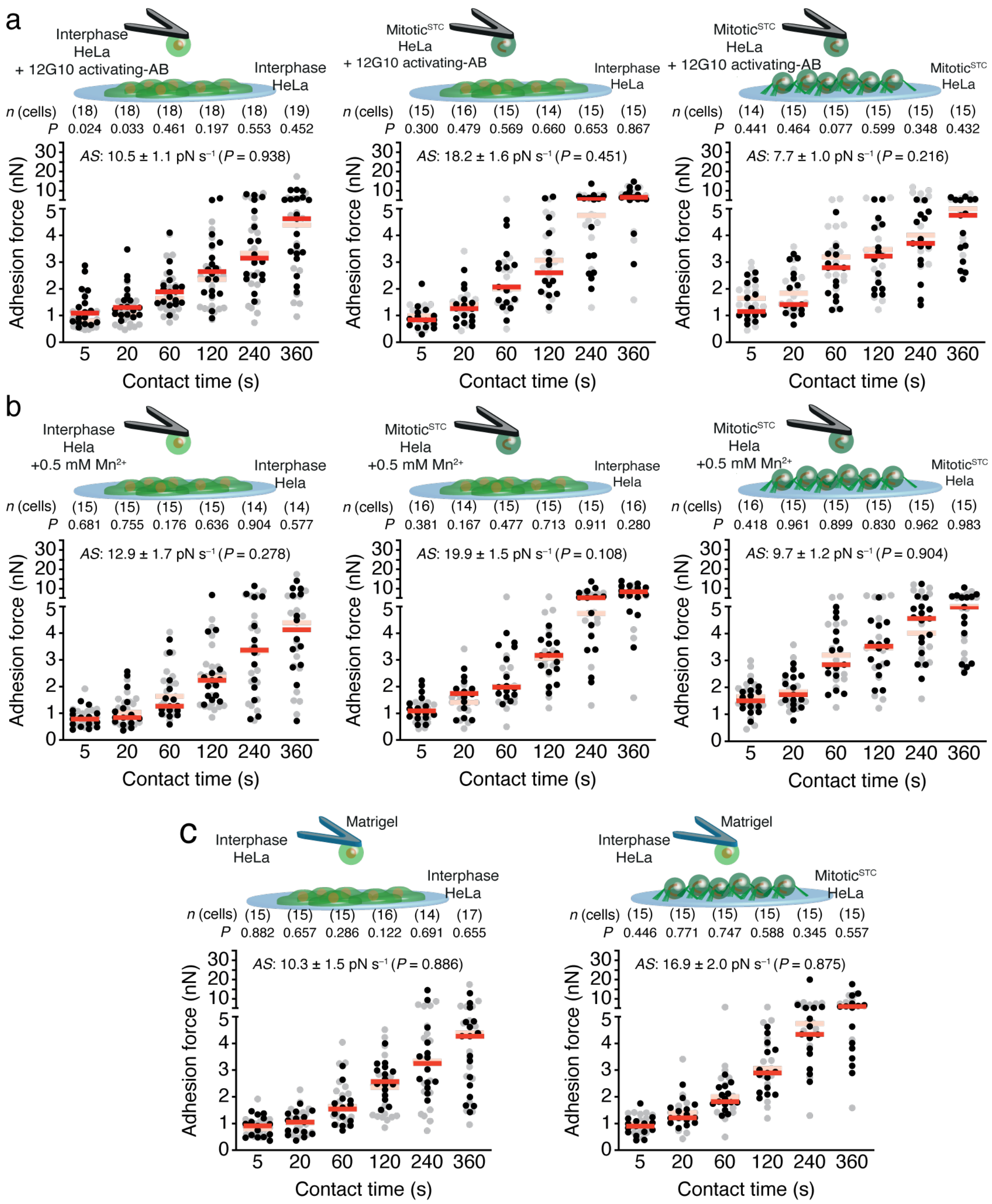

Supplementary Fig. 12 | Activation of β1 integrins does not affect cell-cell adhesion. a,b, Cell-cell adhesion forces between two interphase (left), an interphase and a mitotic^{STC} (middle) or two mitotic^{STC} (right) HeLa cells, incubated with the integrin β1 activating-antibody clone 12G10 (**a**) or Mn^{2+} (**b**) at the given contact times. Dots represent adhesion forces between single cantilever-bound and substrate-spread cells, red bars median values and *n*(cells) the number of tested cells. As reference cell-cell adhesion forces established between two control HeLa cells in the respective condition are given in grey (**Fig. 1g**). *P*-values compare adhesion forces of given and the reference data. *AS*-values give the adhesion strengthening rate as the slope (±SE) of a linear fit through adhesion forces for all contact times with *P*-values comparing the *AS*-value with the *AS*-value of the reference data **c**, Cell-cell adhesion forces between two interphase control HeLa cell (left), an interphase and mitotic^{STC} control HeLa cell (right) on Matrigel-coated cantilever and support, respectively. Data representation as described for **c.** Mitotic cells were enriched by 2 µM STC 12 h before and throughout the experiments. *P*-values comparing adhesion forces were calculated using two-tailed Mann-Whitney tests and comparing *AS*-values to given reference data were calculated by two-tailed extra sum of squares F-tests.

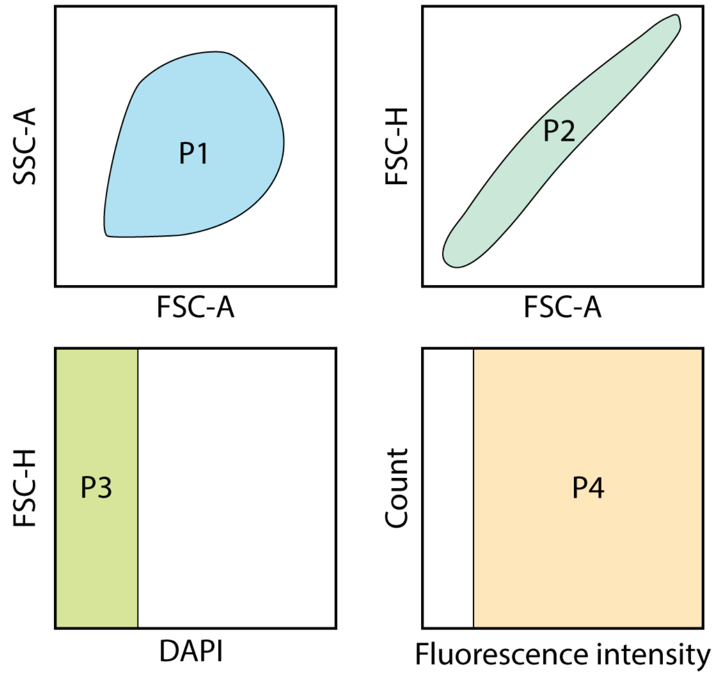

Supplementary Fig. 13 | Gating strategy for flow cytometry experiments. Debris was excluded according to side scatter area (SSC-A) *versus* forward scatter area FSC-A and the depicted population P1 was analyzed further. Doublets were excluded by forward scatter height (FSC-H) *versus* FSC-A and depicted population P2 was further analyzed. Live cells in depicted population P3 were determined by the absence of DAPI signal. The depicted population P4 was used to analyze the median fluorescence intensity of the cell population.

Supplementary Table 1 | Table of *P*-values comparing contact time-dependent spreading area of interphase and mitotic^{STC} HeLa cells shown in **Fig. 1d** using a two-tailed Mann-Whitney test.

**Time (min)**	***P*-value**	**Time (min)**	***P*-value**	**Time (min)**	***P*-value**
2	*0.6200*	22	*0.1649*	42	*0.0041*
4	*0.2593*	24	*0.0530*	44	*0.0041*
6	*0.0530*	26	*0.1282*	46	*0.0041*
8	*0.1282*	28	*0.0728*	48	*0.0041*
10	*0.2086*	30	*0.0530*	50	*0.0041*
12	*0.2593*	32	*0.0262*	52	*0.0082*
14	*0.2593*	34	*0.0175*	54	*0.0727*
16	*0.1649*	36	*0.0111*	56	*0.0424*
18	*0.1282*	38	*0.0111*	58	*0.0424*
20	*0.1649*	40	*0.0111*		

Supplementary Table 2 | Selected short-guide RNAs (sgRNAs) for CRISPR/Cas mediated protein depletion of the adhesome proteins vinculin, talin and kindlin. Given are forward and reverse sequences in capital letters with additional nucleotides in small letters, needed to clone the hybridized sgRNAs into the BbsI-digested backbone plasmid px458.2. Specific exons were analyzed with the online CRISPR design tool (http://crispor.tefor.net) for suitable DNA target sites directly upstream of a requisite 5'-NGG adjacent motif (PAM site) that allows for Cas9 binding and cutting, short-guide RNAs (sgRNA) for respective genes were selected according to their target score, off-target score and efficiency score using the human genome (GRCh38/hg38).

**Protein**	**Exon**	**Forward**	**Reverse**
VCL	1	caccgATGAAACACTGGCATCGCGG	aaacCCGCGATGCCAGTGTTTCATc
TLN1	18	caccgGCTCAAAGTACTGCGCCATC	aaacGATGGCGCAGTACTTTGAGCc
TLN1	29	caccgGCTCGAGCCTCAGGCCGATT	aaacAATCGGCCTGAGGCTCGAGCc
TLN2	37	caccgGGTTTGAGGCGAACGCTGTC	aaacGACAGCGTTCGCCTCAAACCc
TLN2	38	caccgCATTCGCACTGCACGCTCTC	aaacGAGAGCGTGCAGTGCGAATGc
FERMT1	4	caccgCCTGGACCCCATATTTGTCC	aaacGGACAAATATGGGGTCCAGGc
FERMT1	12	caccgGAATCAATACGCCCAATGGA	aaacTCCATTGGGCGTATTGATTCc
FERMT2	3	caccgCATTGGACCTTAGATAAGTA	aaacTACTTATCTAAGGTCCAATGc
FERMT2	12	caccgGTTGTAAGAACTGTCCGCCA	aaacTGGCGGACAGTTCTTACAACc
