# Supplementary material for: In mitosis integrins reduce adhesion to extracellular matrix and strengthen adhesion to adjacent cells
Source: Nat Commun. 2023 Apr 14;14:2143. doi: 10.1038/s41467-023-37760-x (PMC10104879; doi:10.1038/s41467-023-37760-x)
Supplement: Supplementary file 3 — Reporting Summary [file 41467_2023_37760_MOESM3_ESM.pdf]

nature portfolio

Corresponding author(s): Prof. Dr. Daniel J. Müller
Dr. Nico Strohmeyer

Last updated by author(s): Mar 10, 2023

Reporting Summary

Nature Portfolio wishes to improve the reproducibility of the work that we publish. This form provides structure for consistency and transparency in reporting. For further information on Nature Portfolio policies, see our Editorial Policies and the Editorial Policy Checklist.

Statistics

For all statistical analyses, confirm that the following items are present in the figure legend, table legend, main text, or Methods section.

n/a	Confirmed	
☐	☒	The exact sample size (*n*) for each experimental group/condition, given as a discrete number and unit of measurement
☐	☒	A statement on whether measurements were taken from distinct samples or whether the same sample was measured repeatedly
☐	☒	The statistical test(s) used AND whether they are one- or two-sided<br>*Only common tests should be described solely by name; describe more complex techniques in the Methods section.*
☒	☐	A description of all covariates tested
☒	☐	A description of any assumptions or corrections, such as tests of normality and adjustment for multiple comparisons
☐	☒	A full description of the statistical parameters including central tendency (e.g. means) or other basic estimates (e.g. regression coefficient) AND variation (e.g. standard deviation) or associated estimates of uncertainty (e.g. confidence intervals)
☐	☒	For null hypothesis testing, the test statistic (e.g. *F*, *t*, *r*) with confidence intervals, effect sizes, degrees of freedom and *P* value noted<br>*Give P values as exact values whenever suitable.*
☒	☐	For Bayesian analysis, information on the choice of priors and Markov chain Monte Carlo settings
☒	☐	For hierarchical and complex designs, identification of the appropriate level for tests and full reporting of outcomes
☒	☐	Estimates of effect sizes (e.g. Cohen's *d*, Pearson's *r*), indicating how they were calculated

Our web collection on statistics for biologists contains articles on many of the points above.

Software and code

Policy information about availability of computer code

Data collection	For SCFS experiments, a JPK inbuilt data acquisition software was used. For Flow Cytometry, a BD Bioscience inbuilt software was used. For confocal laser scanning microscopy Zeiss (ZenBlue) software was used.
Data analysis	Statistical tests were performed using Prism (GraphPad Software - Version 8.4.3 (471)). Adhesion forces and single molecule interactions used for adhesion probabilities were determined using the JPK data analysis software (JPK Instruments). Adhesion force strengthening was determined using PRISM (Version 8.4.3 (471)). Fluorescence intensities for flow cytometry experiments were determined using the Fortessa software (BD FACSDiva 8.1). Images for confocal imaging were processed using ZEN Blue software (Zeiss), 3D Airyscan processing was performed by using the default settings and image analysis was performed using Fiji.

For manuscripts utilizing custom algorithms or software that are central to the research but not yet described in published literature, software must be made available to editors and reviewers. We strongly encourage code deposition in a community repository (e.g. GitHub). See the Nature Portfolio guidelines for submitting code & software for further information.

Data

Policy information about availability of data

All manuscripts must include a data availability statement. This statement should provide the following information, where applicable:

- Accession codes, unique identifiers, or web links for publicly available datasets
- A description of any restrictions on data availability
- For clinical datasets or third party data, please ensure that the statement adheres to our policy

The data that support the findings and the code to analyze the data are available from the corresponding authors upon request.

Field-specific reporting

Please select the one below that is the best fit for your research. If you are not sure, read the appropriate sections before making your selection.

☒ Life sciences ☐ Behavioural & social sciences ☐ Ecological, evolutionary & environmental sciences

For a reference copy of the document with all sections, see nature.com/documents/nr-reporting-summary-flat.pdf

Life sciences study design

All studies must disclose on these points even when the disclosure is negative.

Sample size	Sample size was not predetermined. The adhesion of at least 10 cells was measured per condition to obtain statistically firm results. We estimated that this number of cells would suffice for biologically relevant differences. For fluorescence intensities, 20'000 cells per sample and a minimum of 4 samples was analyzed to attain statistically firm results. We estimated that this number of cells would suffice for biologically relevant differences. Confocal imaging was performed on at least 5 cells to verify the appearance of any relevant phenotypes.
Data exclusions	No analyzed data was excluded.
Replication	Adhesion forces of at least ten cells per condition and contact time were measured on at least three different days. For confocal imaging at least five cells in at least three different samples were analyzed . Flow cytometry experiments were performed on at least four samples with each containing 20'000 cells on at least three different days.
Randomization	The order of contact times was randomized for every cell and condition. Samples used for flow cytometry were analyzed in a random order and in alternation with other samples tested during the same experiment. Samples were allocated randomly into experimental groups on any given day
Blinding	Blinding was not relevant to this study since measurements (cell adhesion/flow cytometry/microscopy) were analyzed after data acquisition and were therefor not susceptible to experimenter bias.

Reporting for specific materials, systems and methods

We require information from authors about some types of materials, experimental systems and methods used in many studies. Here, indicate whether each material, system or method listed is relevant to your study. If you are not sure if a list item applies to your research, read the appropriate section before selecting a response.

Materials & experimental systems

n/a	Involved in the study	
☐	☒	Antibodies
☐	☒	Eukaryotic cell lines
☒	☐	Palaeontology and archaeology
☒	☐	Animals and other organisms
☒	☐	Human research participants
☒	☐	Clinical data
☒	☐	Dual use research of concern

Methods

n/a	Involved in the study	
☒	☐	ChIP-seq
☐	☒	Flow cytometry
☒	☐	MRI-based neuroimaging

Antibodies

Antibodies used	Anti-talin1/2 AB (ab11188, Abcam),<br>anti-kindlin1 AB (ab68041, Abcam),<br>anti-kindlin2 AB (ab74030, Abcam),<br>anti-vinculin AB (ab18058, Abcam),<br>anti-GAPDH AB (#2118S, Cell Signaling),<br>anti-collagen I AB (ab88147, Abcam, UK),<br>anti-collagen IV AB (PA128534, ThermoFischer, Switzerland),<br>anti-fibronectin AB (ab2413, Abcam, UK),<br>anti-laminin AB (ab256380, Abcam, UK),<br>goat anti-mouse AB (αMouse IgG HRP-conjugated, #1706516, BioRad),<br>goat anti-rabbit AB (αRabbit IgG HRP-conjugated, #1706515, BioRad),<br>anti-CD51 AB (integrin αV, 327909, Biolegend, PE-conjugated),<br>anti-CD49a AB (integrin α1, 328303, Biolegend, PE-conjugated),<br>anti-CD49b AB (integrin α2, 359307, Biolegend, PE-conjugated),<br>anti-CD49c AB (integrin α3, 343803, Biolegend, PE-conjugated),<br>anti-CD49d AB (integrin α4, 304303, Biolegend, PE-conjugated),<br>anti-CD49e AB (integrin α5, 328009, Biolegend, PE-conjugated),

anti-CD49f AB (integrin α6, 313607, Biolegend, Alexa488-conjugated),
anti-CD29 AB (integrin β1, 303015, Biolegend, Alexa488-conjugated),
anti-CD18 AB (integrin β2, 366305, Biolegend, FITC-conjugated),
anti-CD61 AB (integrin β3, 336403, Biolegend, FITC-conjugated),
anti-CD104 AB (integrin β4, 327807, Biolegend, PE-conjugated),
anti-CD29 AB clone 9EG7 (553715, BD Bioscience, un-conjugated),
anti-CDH1 AB (E-Cadherin, 147306, Biolegend, Alexa594-conjugated),
anti-CDH2 AB (N-Cadherin, 350806, Biolegend, PE-conjugated),
anti-CDH5 AB (VE-Cadherin, 348505, Biolegend, PE-conjugated),
PE-conjugated IgG2a antibody (407507, BioLegend),
anti-integrin β1 antibody (AIIB2, DSHB, USA),
anti-integrin β1 antibody (clone 12G10, ab30394, abcam)
anti-rat AlexaFluor 555 (ab150158, Abcam, UK),
anti-rabbit Alexa488 (ab150077, Abcam, UK),
anti-mouse Alexa488 (ab150113, Abcam, UK)

Validation

Anti-talin1/2 AB is according to manufacturer specific to mouse, chicken and chinese hamster with several references given (https://www.abcam.com/talin-1-and-2-antibody-8d4-ab11188.html).
Anti-kindlin1 AB is according to manufacturer specific to rabbit, mouse and human with several references given (https://www.abcam.com/kindlin-1-antibody-ab68041.html).
Anti-kindlin2 AB is according to manufacturer specific to rabbit and human with several references given (https://www.abcam.com/kindlin-2-antibody-ab74030.html).
Anti-vinculin AB is according to manufacturer specific to rabbit, mouse, rat and human with several references given (https://www.abcam.com/vinculin-antibody-epr19579-ab207440.html).
Anti-GAPDH AB is according to manufacturer specific to rabbit, mouse, rat, monkey, bovine, pig and human with several references given (https://www.cellsignal.com/products/primary-antibodies/gapdh-14c10-rabbit-mab/2118).
Goat anti-mouse AB is according to manufacturer specific to mouse and human IgG with several references given (https://www.bio-rad.com/en-ch/sku/1706516-goat-anti-mouse-igg-h-l-hrp-conjugate?ID=1706516#).
Goat anti-rabbit AB is according to manufacturer specific to rabbit and human IgG with several references given (https://www.bio-rad.com/en-ch/sku/1706515-goat-anti-rabbit-igg-h-l-hrp-conjugate?ID=1706515#).
Anti-CD51 AB is according to manufacturer specific to mouse and human with several references given (https://www.biolegend.com/en-us/search-results/pe-anti-human-cd51-antibody-4107).
Anti-CD49a AB is according to manufacturer specific to mouse, human, african green, baboon, cynomolgus and rhesus with several references given https://www.biolegend.com/en-us/products/pe-anti-human-cd49a-antibody-5067).
Anti-CD49b AB is according to manufacturer specific to mouse and human with several references given (https://www.biolegend.com/en-us/products/pe-anti-human-cd49b-antibody-8881).
Anti-CD49c AB is according to manufacturer specific to mouse and human with several references given (https://www.biolegend.com/en-us/products/pe-anti-human-cd49c-integrin-alpha3-antibody-6052).
Anti-CD49d AB is according to manufacturer specific to mouse, human, african green, baboon, cat (feline), cattle (bovine, cow), chimpanzee, common marmoset, cynomolgus, dog (canine), horse (equine), rhesus, sheep (ovine) and squirrel monkey with several references given (https://www.biolegend.com/en-us/products/pe-anti-human-cd49d-antibody-584).
Anti-CD49e AB is according to manufacturer specific to mouse, human, african green, baboon, cynomolgus and rhesus with several references given (https://www.biolegend.com/en-us/products/pe-anti-human-cd49e-antibody-4102).
Anti-CD49f AB is according to manufacturer specific to rat, human, african green, mouse, baboon, capuchin monkey, cat (feline), cattle (bovine, cow), chimpanzee, cynomolgus, dog (canine), horse (equine), rabbit (lapine), rhesus, sheep (ovine) and swine (pig, porcine) with several references given (https://www.biolegend.com/en-us/products/alexa-fluor-488-anti-human-mouse-cd49f-antibody-3289).
Anti-CD29 AB is according to manufacturer specific to mouse, human, african green, baboon, cattle (bovine, cow), cynomolgus, dog (canine), (horse reactivity) and rhesus with several references given (https://www.biolegend.com/en-us/products/alexa-fluor-488-anti-human-cd29-antibody-3257).
Anti-CD18 AB is according to manufacturer specific to mouse and human with several references given (https://www.biolegend.com/en-us/products/fitc-anti-human-cd18-antibody-11721).
Anti-CD61 AB is according to manufacturer specific to mouse, human, african green, baboon, cynomolgus and rhesus with several references given (https://www.biolegend.com/en-us/products/fitc-anti-human-cd61-antibody-5338).
Anti-CD104 AB is according to manufacturer specific to mouse and human with several references given (https://www.biolegend.com/en-us/products/pe-anti-human-cd104-antibody-4121).
Anti-CD29 AB clone 9EG7 is according to manufacturer specific to mouse with several references given (https://www.bdbiosciences.com/en-us/products/reagents/flow-cytometry-reagents/research-reagents/single-color-antibodies-ruo/purified-rat-anti-mouse-cd29.553715).
Anti-CDH1 AB is according to manufacturer specific to mouse, human, rat, dog (canine) and swine (pig, porcine) with several references given (https://www.biolegend.com/en-us/products/alexa-fluor-594-anti-mouse-human-cd324-e-cadherin-antibody-9431).
Anti-CDH2 AB is according to manufacturer specific to mouse and human with several references given (https://www.biolegend.com/en-us/products/pe-anti-human-cd325-n-cadherin-antibody-6982).
Anti-CDH5 AB is according to manufacturer specific to mouse and human with several references given (https://www.biolegend.com/en-us/products/pe-anti-human-cd144-ve-cadherin-antibody-6615).
PE-conjugated IgG2a antibody is according to manufacturer specific to mouse and rat with several references given (https://www.biolegend.com/en-us/products/pe-anti-rat-igg2a-10466).
Anti-integrin β1 antibody AIIB2 is according to manufacturer specific to bovine, canine, human, mouse and sheep with several references given (https://dshb.biology.uiowa.edu/AIIB2).
Anti-integrin β1 antibody 12G10 is according to manufacturer specific to human with several references given (https://www.abcam.com/integrin-beta-1-antibody-12g10-ab30394.html).
Anti-rat AlexaFluor 555 is according to manufacturer specific to rat IgG with several references given (https://www.abcam.com/goat-rat-igg-hl-alexa-fluor-555-ab150158.html).
anti-collagen I AB is according to manufacturer specific to Mouse, Human and Recombinant fragment with several references given (https://www.abcam.com/collagen-i-antibody-3g3-bsa-and-azide-free-ab88147.html)
anti-collagen IV AB is according to manufacturer specific to Bovine, Human, Mammal, Mouse, Rat with references given (https://

www.thermofisher.com/antibody/product/Collagen-IV-Antibody-Polyclonal/PA1-28534)
anti-fibronectin AB is according to manufacturer specific to Mouse, Human with several references given (https://www.abcam.com/fibronectin-antibody-ab2413.html)
anti-laminin AB is according to manufacturer specific to Mouse, Rat, Human (https://www.abcam.com/laminin-beta-1-antibody-epr23265-32-ab256380.html)

Eukaryotic cell lines

Policy information about cell lines

Cell line source(s)	HeLa (kyoto) cells and HeLa cells expressing MYH9-GFP H2B-mCh were a sub-culture that was brought in Nov 2012 from the A. Hyman Lab, Max-Planck-Institute of Molecular Cell Biology and Genetics, Pfotenhauerstrasse 108, 01307 Dresden, Germany.<br>MDCK cells were brought in 2014 from the Botond Roska Lab, Institute of Molecular and Clinical Ophthalmology Basel, Mittlere Strasse 91, CH-4031 Basel, Switzerland.<br>MDCK cells expressing E-Cadherin GFP were originally gifted by Prof. William James Nelson (School of Medicine, Standford, USA).<br>Fibroblasts were provided by Reinhard Fässler, Max Planck Institute of Biochemistry, Department of Molecular Medicine, 82152 Martinsried, Germany.
Authentication	Parental cell lines were not authenticated and engineered cell lines were authenticated by flow cytometry or western blot.
Mycoplasma contamination	All cell lines were tested negative for mycoplasma contamination.
Commonly misidentified lines<br>(See ICLAC register)	No commonly misidentified cells were used. Cells were routinely characterized for their very specific expression patterns of integrins or adhesome proteins.

Flow Cytometry

Plots

Confirm that:

- [x] The axis labels state the marker and fluorochrome used (e.g. CD4-FITC).
- [x] The axis scales are clearly visible. Include numbers along axes only for bottom left plot of group (a 'group' is an analysis of identical markers).
- [x] All plots are contour plots with outliers or pseudocolor plots.
- [x] A numerical value for number of cells or percentage (with statistics) is provided.

Methodology

Sample preparation	Interphase cells were detached from culture flasks using 0.25% (wt/vol) trypsin/EDTA (Sigma Aldrich) for 2 min at 37°C. Cells were resuspended in culture medium, allowed to recover from trypsin treatment for 30 min. To perform flow cytometry with mitotic cells, 2 µM STC was added to cultured cells 12 h prior to and was present throughout experiments. Mitotic cells were harvested by through washing with medium. For flow cytometry experiments in the presence of Mn2+, cells were incubated with 0.5 mM MnCl2 for 30 min in Dulbecco's Modified Eagle Medium (DMEM, 31966047, Thermo Fisher Scientific), supplemented with 100 U ml–1 penicillin and 100 µg ml–1 streptomycin (15140122, Thermo Fisher Scientific) and with 0.5 mM MnCl2 present throughout the experiment. ~125'000 cells per sample were pelleted and washed twice with ice-cold flow cytometry buffer (PBS supplemented with 2 mM EDTA and, 0.1% BSA (wt/vol)). Flow cytometry experiments with antibody against β1 integrins in an extended conformation (clone 9EG7) were performed with an adjusted flow cytometry buffer (PBS supplemented with 1 mM CaCl2, 1 mM MgCl2 and 3% BSA (wt/vol)). Antibodies against CD51 (integrin αV, 327909, Biolegend, PE-conjugated), CD49a (integrin α1, 328303, Biolegend, PE-conjugated), CD49b (integrin α2, 359307, Biolegend, PE-conjugated), CD49c (integrin α3, 343803, Biolegend, PE-conjugated), CD49d (integrin α4, 304303, Biolegend, PE-conjugated), CD49e (integrin α5, 328009, Biolegend, PE-conjugated), CD49f (integrin α6, 313607, Biolegend, Alexa488-conjugated), CD29 (integrin β1, 303015, Biolegend, Alexa488-conjugated), CD18 (integrin β2, 366305, Biolegend, FITC-conjugated), CD61 (integrin β3, 336403, Biolegend, FITC-conjugated), CD104 (integrin β4, 327807, Biolegend, PE-conjugated), CD29 clone 9EG7 (553715, BD Bioscience, un-conjugated), CDH1 (E-Cadherin, 147306, Biolegend, Alexa594-conjugated), CDH2 (N-Cadherin, 350806, Biolegend, PE-conjugated), CDH5 (VE-Cadherin, 336403, Biolegend, PE-conjugated) were diluted 1:10 in ice-cold flow cytometry buffer. Palleted cells were resuspended in 50 µL flow cytometry buffer containing the respective antibodies and incubated for 1h on ice for all pre-conjugated antibodies. Cells incubated with the CD 29 clone 9EG7 antibody were incubated on ice for 30 min with the primary antibody, washed twice with flow cytometry buffer and incubated with 50 µl of 1:10 PE-conjugated IgG2a antibody (407507, BioLegend). Following antibody incubation cells were washed twice with ice-cold flow cytometry buffer and finally resuspended in 250 µL flow cytometry buffer supplemented with 0.25 µg DAPI (422801, BioLegend) and kept on ice.
Instrument	BD LSRFortessa, BD Bioscience
Software	The fluorescence of individual cells and the median fluorescence intensity was determined using the Fortessa software.

Cell population abundance	After excluding debris and doublets 100% of cells were relevant for the study, as we used cell culture condition (100% purity of cells of interest).
Gating strategy	The flow cytometry data was gated according to forward and side scatter to exclude debris and doublets, as well as to the DAPI signal to exclude remaining dead fibroblasts.

☐ Tick this box to confirm that a figure exemplifying the gating strategy is provided in the Supplementary Information.
